# From Lab to Life: Exploring Cutting-Edge Models for Neurological and Psychiatric Disorders

**DOI:** 10.3390/biomedicines12030613

**Published:** 2024-03-08

**Authors:** Masaru Tanaka, László Vécsei

**Affiliations:** 1 HUN-REN-SZTE Neuroscience Research Group, Hungarian Research Network, University of Szeged (HUN-REN-SZTE), Danube Neuroscience Research Laboratory, Tisza Lajos krt. 113, H-6725 Szeged, Hungary; vecsei.laszlo@med.u-szeged.hu; 2 Department of Neurology, Albert Szent-Györgyi Medical School, University of Szeged, Semmelweis u. 6, H-6725 Szeged, Hungary

## 1. Introduction

Neuroscience, neurology, and psychiatry are rapidly evolving fields that aim to understand the complex mechanisms underlying brain function and dysfunction, as well as to develop effective interventions for various neurological and psychiatric disorders [1,2,3]. Recent advances in molecular biology, genetics, epigenetics, pharmacology, and neuroimaging have provided new insights into the etiology, pathophysiology, diagnosis, and treatment of these disorders [4,5,6,7,8]. However, there are still many challenges and gaps in translating basic research findings into clinical applications and improving the quality of life of patients and their families [9,10,11]. One pivotal area of interest within these disciplines is neuroplasticity, the brain’s remarkable ability to reorganize and adapt throughout life [12,13,14,15,16]. Neuroplasticity encompasses various mechanisms, including synaptic plasticity, neurogenesis, and alterations in neuronal connectivity, which underpin crucial processes such as learning, memory, and recovery from injury or disease [17,18,19,20]. In tandem with understanding neuroplasticity, non-invasive brain stimulation (NIBS) techniques such as transcranial magnetic stimulation (TMS) and transcranial direct current stimulation (tDCS) have emerged as promising therapeutic modalities [21,22,23]. These techniques can modulate neuroplasticity by inducing changes in cortical excitability and connectivity, offering potential avenues for ameliorating symptoms associated with conditions such as depression, schizophrenia (SCZ), and chronic pain [24,25,26]. Research indicates that NIBS holds promise for enhancing cognitive function, alleviating mood disturbances, and reducing pain perception by targeting specific brain regions implicated in these processes [27,28,29]. Moreover, combining NIBS with cognitive training, psychotherapy, or pharmacotherapy may enhance treatment outcomes synergistically [30,31,32,33].

To address these challenges and gaps, this Special Issue invited original research articles and reviews focusing on genetic, epigenetic, environmental, and pharmacological models for neuroscience, neurologic diseases, and psychiatric disorders. We aimed to showcase the latest developments and innovations in bench-to-bedside translational research, as well as highlight the opportunities and limitations of various models and methods [34,35,36]. We also aimed to foster interdisciplinary collaboration and communication among researchers and clinicians working in different fields and domains. The Special Issue received 12 high-quality submissions from authors across the world, covering a wide range of topics and disorders, such as Alzheimer’s disease, Parkinson’s disease (PD), Huntington’s disease, SCZ, bipolar disorder, depression, anxiety, autism, addiction, and pain. The articles presented novel findings and perspectives on the molecular and cellular mechanisms, genetic and epigenetic factors, environmental influences, pharmacological interventions, and biomarkers of these disorders, complementing previous research [37,38,39]. The articles also discussed the challenges and future directions of translational research in neuroscience, neurology, and psychiatry. Below, we briefly summarize the main contributions and implications of each article.

## 2. Special Issue Articles

### 2.1. Neurostimulation and Neuroimaging Techniques for Neurological and Psychiatric Disorders

Neuroimaging techniques and brain stimulation such as functional magnetic resonance imaging (fMRI), positron emission tomography/magnetic resonance imaging (MRI) (PET/MRI), electroencephalography, and TMS are powerful tools for exploring the brain mechanisms underlying language processing and recovery in various neurological and psychiatric disorders [40,41,42,43,44]. These techniques can measure hemodynamic, metabolic, and electrophysiological changes in the brain regions involved in language function, as well as modulate their activity and connectivity [42,45,46]. By applying these techniques to different populations, such as patients with fMRI-related anxiety, epilepsy, or aphasia, researchers can gain insights into the factors that affect language performance and plasticity and develop novel interventions to enhance language rehabilitation [47,48,49].

Rassler et al. investigate how healthy subjects cope with anxiety during fMRI scans by analyzing their heart rate, respiration, and brain activity [50]. The authors found that different subjects use different strategies, such as activating a neural pacemaker or entraining brain oscillations with respiration, to modulate their anxiety level. The article also discusses the implications of these findings for understanding the neural mechanisms of anxiety and its treatment. Papageorgiou et al. reviewed the current state and challenges of aphasia rehabilitation, a field that aims to restore language functions after stroke [51]. This article discusses how translational neuroscience, which bridges basic science and clinical practice, can provide insights into the neural mechanisms of neuroplasticity and language recovery. Additionally, it suggests that domain-general cognitive processes support language, which implies that non-linguistic factors may affect aphasia treatment outcomes. The article concludes that a multidisciplinary and translational approach is needed to advance the knowledge and practice of aphasia rehabilitation.

Borbély et al. evaluated the concordance between PET/MRI and electroclinical data in the presurgical evaluation of patients with epilepsy [52]. Their study found that PET/MRI had a high concordance rate with electroclinical data, suggesting that PET/MRI could be a valuable tool in the presurgical evaluation of patients with epilepsy. The study highlights the potential of PET/MRI in improving the accuracy of presurgical evaluation and reducing the risk of complications associated with epilepsy surgery. The findings of this study could have significant implications for the management of patients with epilepsy, particularly those who are candidates for surgery. de Albuquerque et al. investigated the effect of a single application of cerebellar transcranial direct current stimulation (c-tDCS) on motor skill acquisition in PD [53]. The pilot study found that a single application of c-tDCS failed to enhance motor skill acquisition in the condition. The study highlights the need for further research to determine the optimal parameters and duration of c-tDCS to improve motor skill acquisition in the disease. The findings of this study could have significant implications for the development of new therapeutic strategies for PD.

### 2.2. Antioxidant and Anti-Inflammatory Therapies for Neurologic Diseases and Stroke

Neurological disorders, such as stroke, migraine, and epilepsy, are characterized by impairments in brain function and structure, which can have an impact on patients’ quality of life and survival [54,55,56,57]. Finding effective therapeutic interventions to prevent or treat these disorders presents a significant challenge for biomedical research. In this regard, three articles published in this Special Issue investigate the effects of various pharmacological or hormonal treatments on brain function and structure in animal models or human patients suffering from various neurological disorders [58,59,60]. They use a variety of methods to assess the outcomes of the interventions, including biochemical assays, behavioral tests, and neuroimaging techniques [61,62,63]. Their findings shed new light on the mechanisms and potential benefits of these interventions for the prevention and treatment of neurological disorders.

Inoue et al. investigated the potential of sedation therapy in intensive care units (ICUs) to combat oxidative stress by harnessing the power of antioxidants [58]. The research aimed to determine whether common sedatives, such as propofol, thiopental, and dexmedetomidine, have direct free radical scavenging activity. The study identified the direct radical-scavenging activity of various sedatives used in clinical settings and reported a representative case of traumatic brain injury wherein thiopental administration demonstrated antioxidant effects. The findings suggest the potential for the redevelopment of sedatives containing thiopental as an antioxidant therapy, highlighting the importance of further research in this area. The study provides valuable insights into the potential dual benefits of sedatives in ICUs, serving as both sedative agents and antioxidants, which could have significant implications for the management of critically ill patients requiring sedation in ICUs.

Chen et al. used a rat model to investigate the ability of M4P, a TRPM4-blocking antibody, to protect the cerebral vasculature during delayed stroke reperfusion [59]. The study found that M4P reduced mortality rates and infarct volume, improved vascular integrity, and improved cerebral blood flow and functional recovery after delayed stroke reperfusion. These findings suggest that TRPM4-blocking antibodies have therapeutic potential in reducing vascular injury associated with delayed stroke reperfusion, opening up a promising avenue for the development of stroke therapies. Spekker et al. investigated the effect of estradiol treatment on the behavioral and molecular changes induced by repetitive trigeminal activation in a migraine rat model [60]. These changes were found to be enhanced by estradiol treatment, suggesting that estradiol may have a modulatory effect on the pathophysiology of migraine. The study sheds light on the potential role of estradiol in migraine-related mechanisms, emphasizing its significance in migraine research. These studies provide valuable insights into novel treatment approaches for neurological disorders, emphasizing the need for further research in these areas [64].

### 2.3. Genetic and Epigenetic Factors in Neurologic Diseases and Stroke

Stroke is a neurological condition that arises when the flow of blood to a specific area of the brain is disrupted, resulting in brain injury and the impairment of multiple functions [65,66,67]. Stroke is a significant contributor to mortality and impairment on a global scale, necessitating urgent biomedical research to identify effective interventions and preventive measures [68,69,70]. In this context, two articles explore the role of human umbilical cord blood cells, which are a rich source of stem cells and growth factors, in the context of stroke [71,72]. Salafutdinov et al. evaluated the biosafety of human umbilical cord blood mononuclear cells transduced with an adenoviral vector containing human vascular endothelial growth factor cDNA in vitro [71]. The study assessed the transduction efficacy, transgene expression, transcriptome analysis, and secretome profiling of genetically modified cells, yielding valuable insights into their safety and potential applications. The findings add to our understanding of the biosafety aspects of this cellular modification, which is important for the development of new therapeutic strategies. Ikonnikova et al. used a genetic association study and machine learning to investigate platelet reactivity differences in aspirin-treated patients with acute ischemic stroke [72]. The study sought to understand the contribution of genetic features to laboratory aspirin resistance as measured by platelet aggregation, thereby providing insights into the genetic variations that influence platelet reactivity in the context of ischemic stroke and aspirin treatment. The study’s findings add to ongoing efforts to understand the genetic determinants of aspirin resistance, a major concern in ischemic stroke care. These studies offer valuable knowledge about the safety and potential uses of genetically modified cells and the genetic factors that affect platelet reactivity in the context of ischemic stroke and aspirin treatment. They emphasize the need for additional research in these fields.

### 2.4. Physical and Mental Health Interactions in Psychiatric Disorders

Physical health and mental health are closely intertwined and affect each other in various ways [73,74,75]. For example, physical illnesses can increase the risk of developing mental disorders, and vice versa [76,77,78]. To better understand and intervene in these complex and bidirectional relationships, a translational and multidisciplinary approach is needed that bridges the gap between basic and clinical research and incorporates different perspectives and methods from various disciplines [79,80,81]. In this context, the three articles published in this Special Issue of *Biomedicines* adopt such an approach and use various methods and data sources, such as epidemiological studies, randomized controlled trials, meta-analyses, systematic reviews, and clinical guidelines, to provide evidence-based and comprehensive insights into the interactions between physical health and mental health in different populations and contexts [82,83,84]. Their results have important implications for the prevention and treatment of various neurological and psychiatric disorders.

Sobolewska-Nowak et al. investigated the relationship between depression and cardiovascular disease, focusing on common risk factors such as obesity, diabetes, and physical inactivity. The study emphasizes the importance of interdisciplinary collaboration and the incorporation of depression screening into the treatment of cardiac conditions, highlighting the bidirectional relationship between these health issues and the need to address mental health in cardiovascular care [82]. The study by Festa et al. highlights the positive impact of physical activity on cognition across all age groups, emphasizing the benefits of exercise on attention, memory, and executive functions. The study emphasizes the importance of better understanding the mechanisms underlying these effects, as well as the development of appropriate intervention programs based on age and comorbidity, in order to maximize the cognitive benefits of physical activity [83].

The study by De Micheli et al. investigated the relationship between physical health and the transition to psychosis in people at clinically high risk [84]. The research highlights the importance of monitoring physical health outcomes in individuals at clinically high risk for psychosis, emphasizing the need for public health strategies to promote physical health in this population. The study underscores the bidirectional relationship between physical and mental health, highlighting the need for integrated care to improve outcomes in individuals at clinically high risk for psychosis. These studies advocate for integrated care and the promotion of healthy behaviors to improve overall health outcomes, recognizing the interconnectedness of physical and mental health.

## 3. Conclusions

Neurological and psychiatric disorders are among the most prevalent and debilitating conditions that affect millions of people worldwide [85,86,87]. Despite the advances in neuroscience and neurology, the etiology and pathogenesis of these disorders remain largely unknown, and contemporary treatments are often inadequate or associated with adverse effects [88,89,90,91]. Therefore, there is an urgent need to develop new and effective strategies to prevent, diagnose, and treat these disorders [92,93,94]. To achieve this goal, it is essential to establish reliable and relevant models that can recapitulate the complex interactions between the genetic, epigenetic, environmental, and pharmacological factors that contribute to the onset and progression of these disorders [95,96,97]. Moreover, it is important to identify novel targets and mechanisms that can modulate the molecular and functional changes that occur in the brain and peripheral tissues of patients with these disorders [98,99,100,101,102].

This Special Issue on Genetic, Epigenetic, Environmental, and Pharmacological Models for Neuroscience, Neurologic Diseases, and Psychiatric Disorders: Advancement in Bench-to-Bedside Translational Research has presented a collection of 12 articles that cover a wide range of topics and methods in the field of neuroscience and neurology. The articles have demonstrated the importance and challenges of developing and validating animal and cellular models that can mimic the complex pathophysiology and phenotypes of human neurological and psychiatric disorders. The articles have also highlighted the potential of novel pharmacological and non-pharmacological interventions that can modulate the molecular and functional alterations underlying these disorders. The Special Issue has provided valuable insights and perspectives for advancing translational research from basic science to clinical applications. We hope that this Special Issue will stimulate further research and collaboration among researchers and clinicians who share the common goal of improving the diagnosis, prevention, and treatment of neurological and psychiatric disorders. We also hope that this Special Issue will inspire new ideas and innovations that can bridge the gap between bench and bedside and ultimately benefit patients and society. We thank all the authors and reviewers for their contributions to this Special Issue, and we invite the readers to explore the diverse and exciting topics that are presented in this collection.

## Data Availability

Data sharing is not applicable to this article.

## Abbreviations

c-tDCS	Cerebellar transcranial direct current stimulation.
fMRI	Functional magnetic resonance imaging.
ICUs	Intensive care units.
MRI	Magnetic resonance imaging.
PD	Parkinson’s disease.
PET	Positron emission tomography.
SCZ	Schizophrenia.
tDCS	Transcranial direct current stimulation.
TMS	Transcranial magnetic stimulation.
